# Production of raspberry ketone by redirecting the metabolic flux to the phenylpropanoid pathway in tobacco plants

**DOI:** 10.1016/j.mec.2021.e00180

**Published:** 2021-07-27

**Authors:** Takao Koeduka, Sachiho Takarada, Koya Fujii, Akifumi Sugiyama, Kazufumi Yazaki, Masahiro Nishihara, Kenji Matsui

**Affiliations:** a Graduate School of Sciences and Technology for Innovation, Yamaguchi University, Yoshida 1677-1, Yamaguchi, Yamaguchi, 753-8515, Japan; b Research Institute for Sustainable Humanosphere (RISH), Kyoto University, Uji, Kyoto, 611-0011, Japan; c Iwate Biotechnology Research Center, Kitakami, Iwate, 024-0003, Japan

**Keywords:** Raspberry ketone, Volatiles, Anthocyanin, Metabolic switching, Glycosides, Metabolic pathway

## Abstract

Raspberry ketone is one of the characteristic flavors of raspberry fruits, and it is an important and expensive ingredient in the flavor and fragrance industries. It is present at low levels in plant tissues, and its occurrence is limited to a few taxa. In this context, the stable production of nature-identical raspberry ketone using heterologous synthesis in plants hosts has recently garnered the attention of plant biochemists. In this study, we demonstrate the rational switching of the metabolic flow from anthocyanin pigments to volatile phenylbutanoid production via the phenylpropanoid pathway. This shift led to the efficient and stable production of raspberry ketone and its glycosides via heterologous expression of the biosynthetic enzymes benzalacetone synthase (BAS) and raspberry ketone/zingerone synthase 1 (RZS1) in the transgenic tobacco (*Nicotiana tabacum* ‘Petit Havana SR-1’). Additionally, we achieved improved product titers by activating the phenylpropanoid pathway with the transcriptional factor, production of anthocyanin pigment 1 (PAP1), from *Arabidopsis thaliana*. We further demonstrated another metabolic-flow switching by RNA interference (RNAi)-mediated silencing of chalcone synthase (CHS) to increase pathway-intermediate *p*-coumaroyl-CoA in transgenic tobacco for raspberry-ketone production. The redirection of metabolic flux resulted in transgenic lines producing 0.45 μg/g of raspberry ketone and 4.5 μg/g, on the fresh weight basis, of its glycosides in the flowers. These results suggest that the intracellular enforcement of endogenous substrate supply is an important factor while engineering the phenylpropanoid pathway. This strategy might be useful for the production of other phenylpropanoids/polyketides that are produced via the pathway-intermediate *p*-coumaroyl-CoA, in tobacco plants.

## Highlights

• Overexpression of *BAS* and *RZS1* enabled *de novo* production of raspberry ketone in transgenic tobacco.
• Intracellular enforcement of endogenous *p*-coumaroyl-CoA supply improved raspberry-ketone production.
• The metabolic flow switching can produce raspberry ketone and its glycosides with a titer of 0.45 and 4.5 μg/gFW, respectively.

## 1 Introduction

Raspberry ketone [4-(4-hydroxyphenyl)butan-2-one] is an important characteristic aroma component in ripe raspberries (*Rubus idaeus*) (Aprea et al., 2015). This volatile compound is also found in several other plant species in an organ-specific manner, such as rhubarb roots, pine needles, and in the orchid *Bulbophyllum* flowers, while its amount in these species is very low, even from raspberries (1–4 mg/kg fruit) (Higuchi and Donnelly, 1977; Larsen and Poll, 1990; Larsen et al., 1991; Nishida et al., 1993; Abe et al., 2001). Therefore, naturally-derived raspberry ketone is one of the most expensive flavor compounds (US$ 3000/kg), which is as valuable a flavoring agent as natural vanillin (Lee et al., 2016). In addition to its flavoring applications, raspberry ketone has been utilized in the cosmeceutical industry for its weight-loss and skin-whiting properties (Morimoto et al., 2005; Lin et al., 2011).

**Fig. 1:**
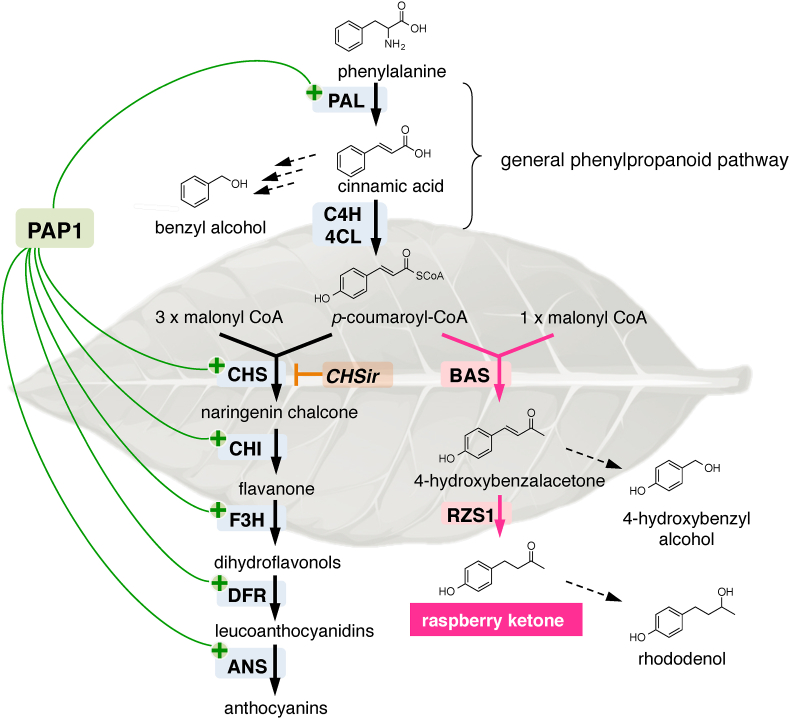
**Engineering of raspberry-ketone production in *Nicotiana tabacum*.** Phenylpropanoid pathway that leads to the production of anthocyanins and raspberry-ketone. Overexpression of benzalacetone synthase (BAS) and raspberry ketone/zingerone synthase 1 (RZS1; pink lines) induces the production of high amounts of raspberry ketone and its derivatives in transgenic tobacco. Co-expression of the PAP1 transcription factor (green lines) and RNAi-mediated suppression of chalcone synthase (CHSir; orange lines) increase the levels of raspberry ketone. Dashed lines indicate the putative pathways. Abbreviations: PAL, phenylalanine ammonia-lyase; C4H, cinnamate 4-hydroxylase; 4CL, 4-coumarate-CoA ligase; CHI, chalcone isomerase; F3H, flavonoid 3′-hydroxylase; DFR, dihydroflavonol 4-reductase; ANS, anthocyanidin synthase. (For interpretation of the references to color in this figure legend, the reader is referred to the Web version of this article.)

The biosynthetic pathway leading to raspberry ketone production shares a common metabolic pathway with anthocyanin pigments, branching from *p*-coumaroyl-CoA, which is biosynthesized via the general phenylpropanoid pathway from phenylalanine by phenylalanine ammonia-lyase (PAL), cinnamate 4-hydroxylase (C4H), and 4-coumarate-CoA ligase (4CL; Fig. 1). The C_6_–C_4_ skeleton of raspberry ketone is synthesized from *p*-coumaroyl-CoA via sequential two-step reactions catalyzed by a Type-III polyketide synthase, benzalacetone synthase (BAS) (Abe et al., 2001). BAS catalyzes the formation of 4-hydroxybenzalacetone through the decarboxylative condensation of *p*-coumaroyl-CoA with malonyl-CoA, followed by hydrolysis and decarboxylation. The resulting intermediate product, 4-hydroxybenzalacetone, is reduced to raspberry ketone by the NADPH-dependent reductase, raspberry ketone/zingerone synthase 1, RZS1 (Koeduka et al., 2011).

The pathway and enzymes involved in raspberry-ketone biosynthesis have been well-characterized, and many studies have been performed, aiming to produce raspberry ketone in heterologous systems using microbes, such as *Escherichia coli*, *Saccharomyces cerevisiae*, and *Corynebacterium glutamicum*, by microbial metabolic engineering (Lee et al., 2016; Wang et al., 2019; Milke et al., 2020). However, there are few reports about metabolic engineering using plant hosts for the production of valuable aroma compounds, such as raspberry ketone. Indeed, plants provide an excellent platform for bio-production of volatile organic compounds because, unlike microbial cells, plant cells have the ability to accumulate a broad range of specialized metabolites as the corresponding glycosides by the activity of endogenous glycosyltransferases. For example, transgenic petunia overexpressing *S*-linalool synthase (*Lis*) gene from *Clarkia breweri* produced the nonvolatile *S*-linalyl-β-D-glucopyranoside (Lücker et al., 2001). Similarly, expression of the strawberry dual linalool/nerolidol synthase (*FaNES1*) gene in *Arabidopsis* under control of a constitutive promoter resulted in the accumulation of linalool glycosides (Aharoni et al., 2003). Therefore, it seems that the plant ability for glycosylation is suitable for trapping volatiles in the cells, and are thus an attractive target for *de novo* production of useful biological compounds, including raspberry ketone. More importantly, plants need only three fundamental elements, i.e., carbon dioxide, water, and sunlight for the production of those phenolic compounds, without need for precursor addition or a culture medium for their growth, conveniently fitting the context of Sustainable Development Goals (SDGs).

In this study, we attempted to express two genes *Rhuem palmatum benzalacetone synthase* (*RpBAS*) and *Rubus idaeus raspberry ketone/zingerone synthase 1* (*RiRZS1*) for the production of raspberry ketone in tobacco plants (*Nicotiana tabacum* ‘Petit Havana SR-1’). As a strategy for increasing the levels of raspberry ketone and its glycosides, *RpBAS*-*RiRZS1*-overexpressing plants were genetically crossed with *Arabidopsis thaliana production of anthocyanin pigment 1* (*AtPAP1*)-overexpressing plants, a master gene of the phenylpropanoid pathway actively responsible for producing anthocyanin pigments in plants, or with *chalcone synthase* (*CHS*)-suppressing plants bearing white flowers, owing to the limited precursors for anthocyanin production by RNA interference (RNAi). The co-expression of *RpBAS* and *RiRZS1* with *AtPAP1* by cross-pollination increased the accumulation of raspberry ketone and its glycosides in tobacco plants by redirecting the metabolic flux toward the biosynthesis of raspberry-ketone.

## 2 Materials and methods

### 2.1 Plant materials and growth conditions

Tobacco plants harboring *pro35S:RiRZS1-RpBAS*; *pro35S:AtPAP1* (Mitsunami et al., 2014); *pro35S:CHSir* (Nakatsuka et al., 2007); and wildtype tobacco plants were grown in a growth room under a 14 h/10 h (L/D) photoperiod at 25°C.

### 2.2 Vector construction

*RiRZS1*, NCBI Accession no. JN166691, was obtained as previously described by Koeduka et al. (2011). *RpBAS*, NCBI Accession no. AF326911, was synthesized from GeneArt (Thermo Fisher Scientific, Waltham, MA, USA). For heterologous expression of *RpBAS* and *RiRZS1* in tobacco plants, the full-length cDNAs were amplified with KOD FX Neo polymerase (Toyobo Co. Ltd., Osaka, Japan) using gene-specific primers (Supplementary Table 1). The resulting PCR products of *RpBAS* and *RiRZS1* containing flanking *Nde*I and *Xho*I or *Sal*I restriction sites were cloned into the *Nde*I and *Sal*I-digested pRI201-AN vector (Takara Bio USA, Mountain View, CA, USA) independently via the intermediate pGEM-T easy TA-cloning vector (Promega, Tokyo, Japan). The *RpBAS* expression cassette containing the CaMV35S promoter with *Arabidopsis* alcohol dehydrogenase 5′-untranslated region and heat shock protein terminator was amplified using PCR and cloned into the *pro35S:RiRZS1* plasmid behind the *RiRZS1* expression cassette by InFusion (Takara Bio USA, Mountain View, CA, USA), according to manufacturer's instructions. The resulting vector carrying *pro35S:RiRZS1-pro35S:RpBAS* was transformed into *Agrobacterium tumefaciens* LBA4404 using electroporation.

### 2.3 Plant transformation

Strain LBA4404 of *A. tumefaciens* harboring vector *pro35S:RiRZS1-pro35S:RpBAS* was used to transform *N. tabacum* ‘Petit Havana SR-1’ by a standard protocol (Horsch et al., 1985). Tobacco seeds were surface sterilized for 5 min with a 10 % (v/v) NaClO solution. Sterilized seeds were rinsed five times with sterile water and subsequently spread on Petri dishes containing solid half-MS medium. Young-leaf discs (0.5–0.7 cm in diameter) from tobacco plants grown in vitro for three weeks were used for infiltration. Tobacco leaf transformation and regeneration were performed as previously described by Horsch et al. (1985). Transgenic plants (*pro35S:RiRZS1-pro35S:RpBAS*; *RB*-OX) were generated under kanamycin (100 mg/L) selection from independent calli. Transgenic plants were transferred into soil and grown in a culture room under a 16/8 h (L/D) photoperiod at 25°C. The resulting transgenic T_1_ seeds were tested for germination on MS medium supplemented with 30 mg/L kanamycin, under 14/10 h (L/D) photoperiod at 25°C. T_2_ seeds harvested from each T_1_ plant showing approximately a 3:1 segregation ratio were screened for kanamycin resistance once more; finally, T_2_ and T_3_ homozygous plant lines were used for further experiments.

### 2.4 Cross-pollination

*Arabidopsis* production of anthocyanin pigment 1 (PAP1) overexpressing tobacco (*pro35S:PAP1*; *PAP1*-OX) Petit Havana SR-1 and RNAi-mediated CHS silencing tobacco (*pro35S:CHSir*; *CHSir*-OX) Petit Havana SR-1 were previously generated (Mitsunami et al., 2014; Nakatsuka et al., 2007) and used to obtain the cross-pollinated lines (*RB*-OX × *PAP1*-OX, named RBP; *RB*-OX × *CHSir*-OX, named RBC). The cross-pollinated RBP lines showed anthocyanin pigmentation on their leaf tissues and these transgenic tobacco plants co-expressing *RiRZS1*, *RpBAS*, and *PAP1* were primarily screened by anthocyanin-pigmented leaf tissues and further selected using PCR screening with genomic DNA. Cross-pollinated RBP and RBC lines were self-pollinated to harvest F_2_ seeds.

### 2.5 RNA extraction and expression analysis

Total RNA was extracted from tobacco leaf tissues using a Plant Total RNA Mini Kit (Chiyoda Science Co., Tokyo, Japan) for RT-PCR. After treatment with DNase using a DNA-free DNA Removal Kit (Thermo Fisher Scientific), cDNAs were synthesized using ReverTra Ace reverse transcriptase (Toyobo Co. Ltd.). The primers used are shown in Supplementary Table 1. Quantitative reverse transcriptase-polymerase chain reaction (qRT-PCR) was performed using the KAPA SYBR Fast qPCR Kit (Nippon Genetics Co., Ltd, Tokyo, Japan) according to the manufacturer's instructions. The tobacco *Elongation Factor 1 alpha* (*EF1a*; GenBank D63396) gene was used to normalize the transcript levels in each sample to those of the reference control. Semi-quantitative reverse transcriptase (sqRT)-PCR was performed using KOD FX Neo polymerase (Toyobo Co. Ltd.) at an initial denaturation of 94°C/3 min, followed by 35 cycles of 98°C/10 s and primer specific annealing and extension at 65°C/30 s and 68°C/30 s, respectively. sqRT-PCR products were separated using agarose gel electrophoresis to visualize the amplified cDNAs. *N. tabacum Actin 9* gene (*NtACT9*; GenBank X69885) was used to normalize transcript levels in each sample as a reference control.

### 2.6 Analysis of volatiles

Apical shoot flowers (300 mg) or leaves (1 g) of transgenic tobacco plants (3 to 4-month-old of T_2_ generation) were ground in liquid nitrogen using mortar and pestle, and the volatiles were extracted with 4 or 10 mL methyl *tert*-butyl ether, respectively, containing 10 μg safrole as an internal standard. The extracts were transferred into glass tubes, sonicated for 10 min, and then centrifuged at 2000 rpm (VC36S, TAITEC Co., Koshigaya City, Japan) for 15 min; supernatants containing volatiles were transferred into clean glass tubes. Then, the extracted samples were concentrated using a vacuum evaporator centrifuge (VC36S, TAITEC) and anhydrous sodium sulfate was used to remove water from the extracts before gas chromatography-mass spectrometry (GC−MS) analysis.

Extracted volatiles were analyzed by GC–MS using a GCMS-QP2010 Plus (Shimadzu, Kyoto, Japan) instrument equipped with a 0.25 mm × 30 m (film thickness of 0.25 μm) DB-5ms column (Agilent, Santa Clara, CA, USA). The column temperature was programmed as follows: 80°C for 2 min, increasing by 10°C/min to 170°C and by 20°C/min to 240°C, with 240°C held for 5 min. Helium carrier gas was delivered at a pressure of 260 kPa. The temperature of the injection port was 180°C. The mass detector was operated in electron-impact mode with an ionization energy of 70 eV. Identification of compounds was performed by comparison of retention times and mass spectra of authentic standards.

### 2.7 Analysis of glycosidically-bound volatiles

Flowers (300 mg) or leaves (1 g) of each transgenic tobacco line were collected and ground in liquid nitrogen. The tissue fine powders were suspended with 3 or 10 mL of 80 % (v/v) methanol and sonicated for 20 min. After centrifugation at 2000 rpm (VC36S, TAITEC) for 15 min, supernatants containing glycosidically-bound volatiles were transferred into clean glass tubes, and methanol was removed using a vacuum evaporator centrifuge (VC36S, TAITEC). The crude glycoside fractions were mixed with 0.3 mL of 10 mM 2-(*N*-morpholino)ethanesulfonic acid (MES)-KOH at pH 5.5 and 0.1 mL of 5 mg/mL β-glycosidase from almond (Sigma-Aldrich, St. Louis, MO, USA) and incubated at 37°C for 12 h with gentle shaking. The formed volatiles were extracted with 4 mL methyl *tert*-butyl ether, containing 10 μg safrole as an internal standard and analyzed by GC–MS as described previously.

### 2.8 Measurement of anthocyanin contents

To measure the anthocyanin contents in the flowers and leaves of transgenic tobacco plants, fully expanded leaves (200 mg) and three flowers from different plants of each transgenic line were collected. Total anthocyanins were extracted with 2 mL of 80 % methanol containing 1 % hydrochloric acid and measured spectrophotometrically at 530 nm of wavelength as described by Nakatsuka et al. (2006).

## 3 Results

### *3.1* *Generation of transgenic tobacco plants overexpressing* RpBAS and RiRZS1

**Fig. 2:**
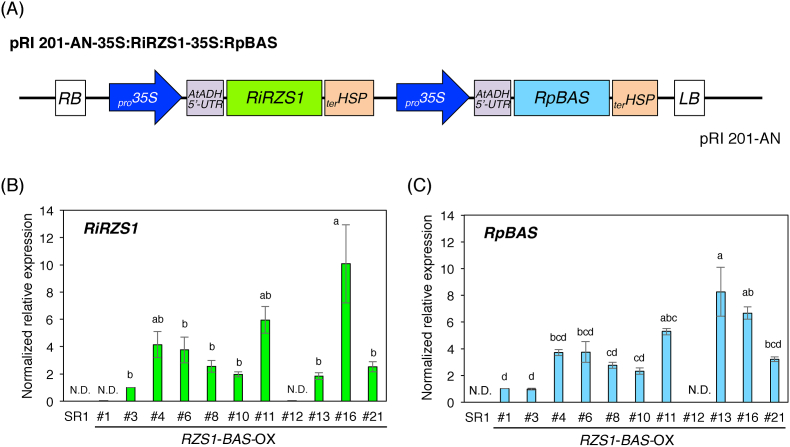
**Transcript analysis of *RZS1* and *BAS* expression in transgenic tobacco (T**_**2**_**generation).** (A) Expression construct of *RZS1* and *BAS* used in this study. (B) The expression levels of *RiRZS1* in *RZS1*-*BAS* overexpressing transgenic tobacco leaves. (C) The expression levels of *RpBAS* in *RZS1*-*BAS*-overexpressing transgenic tobacco leaves. Expression levels were examined using quantitative RT-PCR and normalized to those of the internal reference gene *Elongation Factor 1 alpha* (*EF1a*). Significant differences were identified using one way ANOVA with Fisher's least significance differences test (P < 0.05). N.D., not detected. _pro_35 S, CaMV35S promoter; AtADH 5′-UTR, five prime untranslated region of *A. thaliana* alcohol dehydrogenase; RiRZS1, *Rubus idaeus* raspberry ketone/zingerone synthase 1; RpBAS, *Rheum palmatum* benzalacetone synthase; _ter_HSP, terminator of *A. thaliana* heat shock protein.

To produce raspberry ketone in tobacco plants, an overexpression construct for both *RpBAS* and *RiRZS1* were prepared by insertion downstream of the *CaMV 35S* promoter and *Arabidopsis* alcohol dehydrogenase 5′-untranslated region sequence (*AtADH 5*′*-UTR*), which functions as a translational enhancer in dicotyledonous plant cells, in pRI201-AN binary vector (Fig. 2A). Then, we generated transgenic tobacco plants overexpressing both *RpBAS* and *RiRZS1* by *Agrobacterium*-mediated leaf-disc transformation in accordance with a standard protocol (Horsch et al., 1985). Using kanamycin selection, we obtained more than twenty independent T_0_ transformants. Through PCR analysis of genomic DNA from the transgenic tobacco plants, eleven independent lines (T_0_ generation) harboring both *RpBAS* and *RiRZS1* were selected and we obtained T_1_ generation seeds from each T_0_ transgenic lines. Similarly, we identified T_2_ through T_1_ transgenic lines showing integration of *RpBAS* or *RiRZS1*, or both, using genomic PCR analysis (Supplementary Fig. 1).

**Fig. 3:**
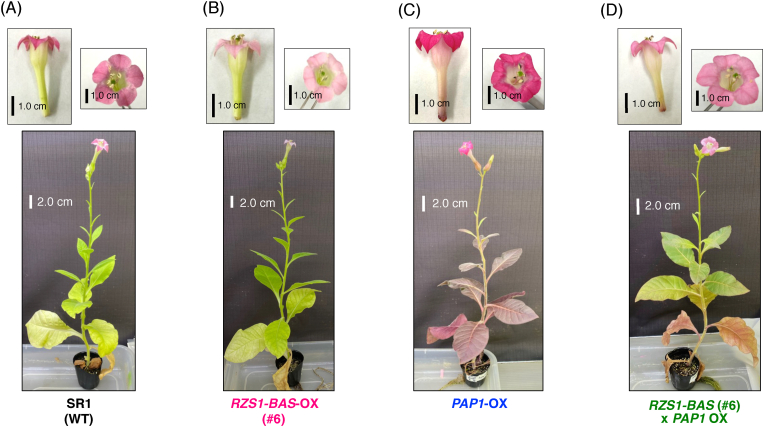
**Phenotype of engineered transgenic tobacco plants at flowering stage (T**_**2**_**generation).** (A) Wildtype tobacco cv. SR1. (B) *RZS1*-*BAS* overexpressing transgenic tobacco. (C) *PAP1* overexpressing transgenic tobacco. (D) *RZS1*-*BAS* and *PAP1* overexpressing transgenic tobacco. Scale bars indicate 1.0 cm (upper panels) and 2.0 cm (lower panels), respectively. RZS1, raspberry ketone/zingerone synthase 1; BAS, benzalacetone synthase; PAP1, production of anthocyanin pigment 1.

**Fig. 4:**
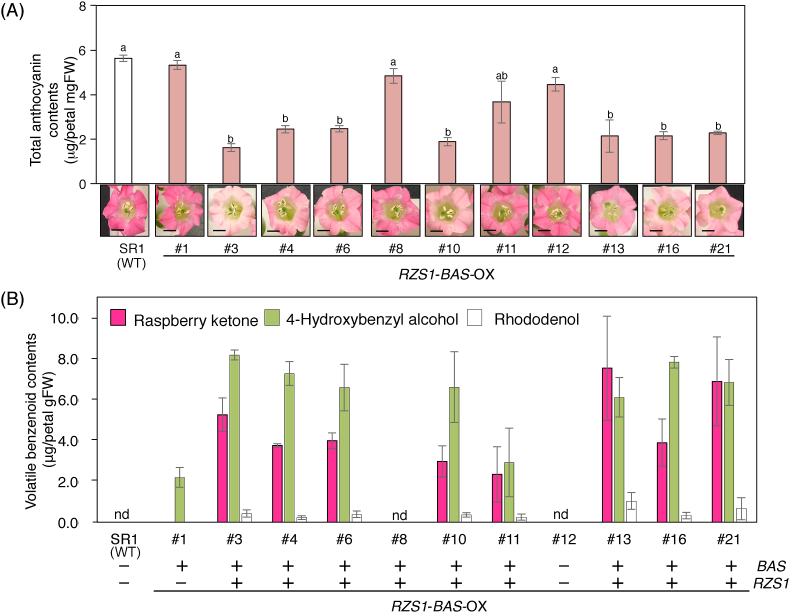
**Anthocyanin accumulation and raspberry-ketone production in petals of *RZS1*-*BAS* overexpressing transgenic tobacco.** (A) Anthocyanin accumulation in petals of *RZS1*-*BAS* overexpressing transgenic tobacco. Total anthocyanin amount in different transgenic tobacco flower petals overexpressing *RZS1* and *BAS* are shown. Photographs show the transgenic flowers used for anthocyanin analysis in this study. Scale bar indicates 1.0 cm. Three flower petals were collected from independent transgenic tobacco plants and total anthocyanin content was quantified under the same condition (n = 3 biological replicates). Significant differences indicated by different lowercase letters were identified using Tukey's HSD tests after one-way ANOVA (*P* <0.05). RZS1, raspberry ketone/zingerone synthase 1; BAS, benzalacetone synthase. (B) Volatile benzenoid contents in the petals of transgenic tobacco overexpressing *RZS1 and BAS*. Raspberry ketone and its related compounds were extracted from three flower petals from a transgenic tobacco plant. Values are presented as means ± standard errors of three different samples. nd, not detected; RZS1, raspberry ketone/zingerone synthase 1; BAS, benzalacetone synthase. The expression of *RZS1* and *BAS* genes in each transgenic tobacco are represented by + and −.

To examine whether the *RpBAS* and *RiRZS1* genes were overexpressed in tobacco plants, we performed qRT-PCR analyses. Among transgenic T_2_ plants, the expression of both *RpBAS* and *RiRZS1* genes was detected in the nine transgenic lines, whereas transgenic line #1 showed the detection of only *RpBAS*, and the expression of both *RpBAS* and *RiRZS1* were undetectable in line #12 (Fig. 2B and C), despite the fact that these two lines showed kanamycin resistance. Further, we checked whether overexpression of *RpBAS* and *RiRZS1* in tobacco plants was associated with significant phenotypic changes in vegetative and reproductive development. Vegetative growth of transgenic plants was morphologically similar to that of control plants (SR1 wildtype plants; Fig. 3, Supplementary Fig. 2), although a clear phenotype of reduced floral pigmentation was observed in some of *RB*-OX transgenic plants (T_2_ generation) compared to wildtype plants. Given that anthocyanin pigments are responsible for flower coloration, anthocyanin content was evaluated using extracts prepared from the mature petals of control wildtype plants and *RB*-OX transgenic plants (Fig. 4A). Consistently with the differences observed in color intensity, the total anthocyanin contents in some *RB*-OX lines (#3, #4, #6, #10, #13, #16, and #21) were approximately 2 to 3-fold lower than that accumulated in control plants. In contrast, there was no significant phenotypic difference in leaf tissues between wildtype (control) and *RB*-OX transgenic plants (Fig. 3 and Supplementary Fig. 2).

**Fig. 5:**
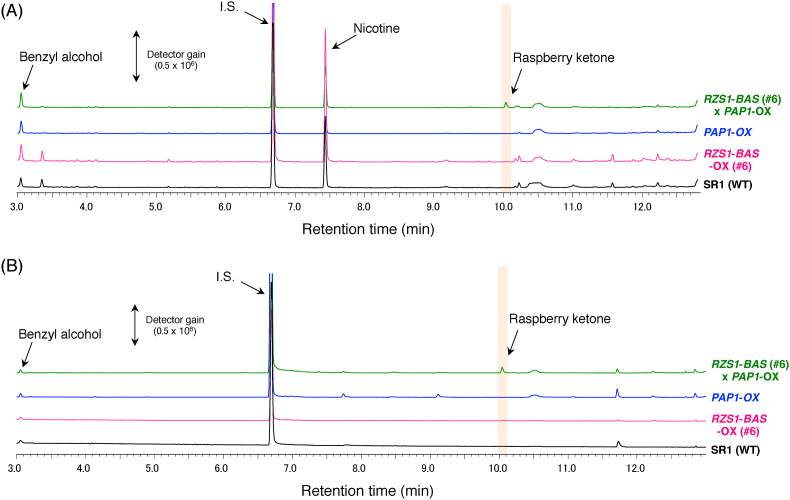
**Detection of VOC aglycones accumulated in three different transgenic tobacco lines.** (A) GC–MS chromatograms of leaf extracts from transgenic tobacco. (B) GC–MS chromatograms of flower extracts from transgenic tobacco. The peaks of each volatile compound were identified by comparing with the retention time and mass spectrum of authentic standards. The peak of raspberry ketone is shaded in orange. (For interpretation of the references to color in this figure legend, the reader is referred to the Web version of this article.)

In addition to the analysis of anthocyanin content, we evaluated the production of raspberry ketone and other volatile compounds in the petal tissues of T_2_ transgenic lines using GC–MS (Fig. 4B and Supplementary Fig. 3). Except for line #8, GC–MS analysis of *RB*-OX transgenic lines showed a significant accumulation of raspberry ketone in the petals, ranging from 2.3 to 7.5 μg/g (average = 4.6 ± 0.6 μg/g) fresh weight (FW); 4-hydroxybenzyl alcohol also accumulated in the petals with an average of 6.5 μg/g FW. In these transgenic lines, a slight accumulation of rhododenol, with an average of 0.5 μg/g FW, was also detected. In contrast, the *RB*-OX transformant #1, which overexpressed only the *BAS* gene, showed a slight accumulation of 4-hydroxybenzyl alcohol, with an average of 2.2 ± 0.5 μg/g FW. Interestingly, raspberry-ketone derivatives were not detected in leaf tissues of any *RB*-OX transgenic plants (Fig. 5A).

### 3.2 Enhanced production of raspberry-ketone derivatives by cross-pollination between RB-OX and PAP1-OX transgenic plants

**Fig. 6:**
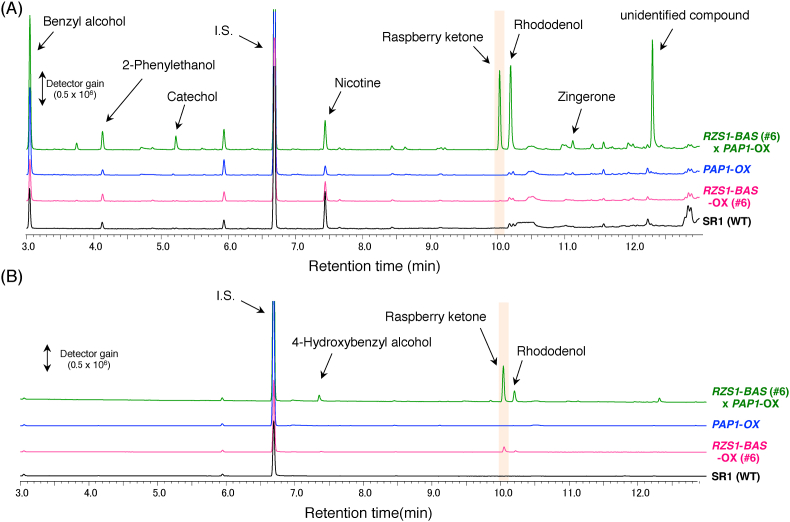
**Detection of raspberry-ketone glycosides and their related compounds in three different transgenic tobacco lines.** (A) GC–MS chromatograms of leaf extracts from transgenic tobacco. (B) GC–MS chromatograms of flower extracts from transgenic tobacco. Glycosidically-bound volatile compounds in different parts of each transgenic tobacco line were subjected to GC-MS analysis after enzymatic hydrolysis by almond β-glycosidase, as described in “Materials and Methods”. The peaks of each volatile compound were identified by comparing with the retention time and mass spectrum of authentic standards. The peak of raspberry ketone is shaded in orange. (For interpretation of the references to color in this figure legend, the reader is referred to the Web version of this article.)

Unfortunately, we failed to detect raspberry ketone in the leaf tissues of *RB*-OX transgenic plants (Fig. 5A). In a previous report, we noted that the overexpression of *AtPAP1* induced the expression of biosynthetic genes encoding PAL, CHS, CHI, F3H, and DFR enzymes in the anthocyanin biosynthetic pathway (Fig. 1) (Mitsunami et al., 2014). This finding suggested that the *AtPAP1* transcriptional factor induces enhanced production of endogenous pathway-intermediate *p*-coumaric acid that can be metabolized into precursors for raspberry-ketone production. To test our hypothesis, we pollinated the flowers of *PAP1*-OX with pollen from *RB*-OX transgenic tobacco plants. The F_2_ seeds produced from cross-pollinated plants were planted and their progenies were primarily screened by visualization of anthocyanin pigments and confirmed by sqRT-PCR analysis (Supplementary Fig. 4). Raspberry ketone and its derivatives present in the leaves and flowers of cross-pollinated F_2_ plants were analyzed by GC–MS. Production of raspberry ketone and its derivatives was detected in both organs (Fig. 5). After enzymatic hydrolysis by almond β-glycosidase, raspberry-ketone glycosides accumulated in the leaves and flowers of cross-pollinated F_2_ plants were analyzed using GC–MS and compared with those of the wildtype, *RB*-OX, and *PAP1*-OX transgenic plants. In the leaves of cross-pollinated F_2_ plants, 2.24 ± 0.18 μg of raspberry ketone glycosides/g FW and 2.29 ± 0.18 μg of rhododenol glycosides/g FW were detected (Fig. 6A, Supplementary Table 2). In contrast, 2.38 ± 0.35 and 4.46 ± 0.21 μg/g FW glycosylated raspberry ketone was detected in the flowers of the *RB*-OX and in cross-pollinated F_2_ plants, respectively, as the predominant compound with the glycosides of 4-hydroxybenzyl alcohol or rhododenol, or both (Fig. 6B, Supplementary Table 3).

The accumulated anthocyanin pigments in the leaves and flowers of the cross-pollinated F_2_ plants were compared with those of the wildtype, *RB*-OX, and *PAP1*-OX transgenic plants (Supplementary Fig. 5). Leaves of the cross-pollinated plants (F_2_) showed higher anthocyanin accumulation levels than those of the *RB*-OX plants but lower than those of the *PAP1*-OX plants. Similar accumulation patterns were observed in the flowers of the cross-pollinated transgenic plants between *RB*-OX and *PAP1*-OX. These results suggest that all transgenes of *RpBAS*, *RiRZS1*, and *AtPAP1* genes were expressed and functioned in the F_2_ cross-pollinated plants through cross-pollination.

### *3.3* *Enhanced production of raspberry-ketone derivatives by cross-pollination between* RB*-OX and* CHSir*-OX transgenic plants*

**Fig. 7:**
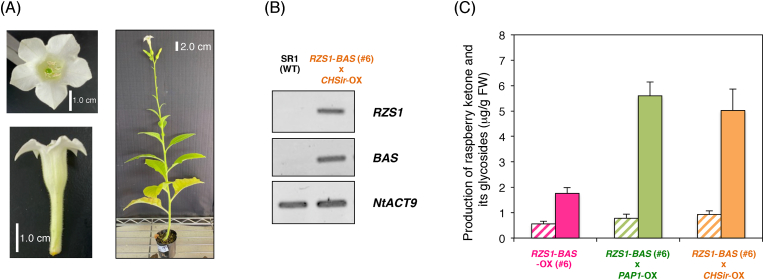
**Accumulated raspberry ketone and its glycosides in *CHSir*-transgenic tobacco flowers.** (A) Phenotype of *RZS1-BAS* (#6) × *CHSir* transgenic tobacco plants. (B) Transgene detection by amplification of *RZS1* and *BAS* in transgenic tobacco plants. (C) Levels of raspberry ketone and its glycosides are shown in transgenic tobacco flowers. The amplification of *BAS*, *RZS1*, and *NtACT9* were analyzed using genomic-DNA PCR. *NtACT9* was used as an internal control. *RZS1*, *raspberry ketone/zingerone synthase 1*; *BAS*, *benzalacetone synthase*; *NtACT9*, *N. tabacum Actin 9*. Shaded and closed bars indicate aglycone and glycosides of raspberry ketone, respectively.

Cross-pollinated *RB*-OX and *PAP1*-OX plants showed enhanced production of raspberry-ketone derivatives. This finding is likely due to the increase in metabolic flux of intermediates in the phenylpropanoid pathway through the transcriptional activation of biosynthetic genes, including *PAL*, by overexpression of *AtPAP1* transcriptional factor (Fig. 1) (Mitsunami et al., 2014). To confirm this hypothesis, we generated cross-pollinated plants of *RB*-OX and *CHSir*-OX, which produced white flowers owing to the decrease in anthocyanin precursors, to redirect the metabolic flux of flavonoid pathway to the raspberry-ketone biosynthetic pathway (Fig. 7). The transgenes of *RpBAS* and *RiRZS1* were confirmed by genomic PCR in the cross-pollinated plants. Moreover, the accumulated levels of raspberry ketone and its glycosides were approximately 1.6- to 3-fold higher (0.93 μg/g FW of raspberry ketone and 5.02 μg/g FW of its glycosides) in the cross-pollinated plants than in the *RB*-OX plants, and the levels were similar to those in cross-pollinated *RB*-OX and *PAP1*-OX plants (0.78 μg/g FW of raspberry ketone and 5.60 μg/g FW of its glycosides). These results suggest that RNAi-mediated suppression of chalcone synthase, which is the branching point between the flavonoid and the phenylpropanoid pathway, increased the flux of the phenylpropanoid pathway, thus increasing substrate flux toward the biosynthesis of raspberry ketone.

## 4 Discussion

In this study, we showed that the co-expression of *RpBAS*, *RiRZS1*, and *AtPAP1* genes resulted in the enhanced production of raspberry ketone and its derivatives in tobacco plants. BAS and RZS are key enzymes leading to the formation of raspberry ketone using *p*-coumaroyl-CoA, the precursor compound which is widely present in plants as an intermediate for numerous aromatic compounds. Therefore, metabolic engineering for raspberry-ketone biosynthesis in transgenic plants using the *BAS* and *RZS* genes seems a rational case of feasible metabolic-flow switching. There have been several attempts to synthesize raspberry ketone in heterologous-expression systems by expressing plant *BAS* or *RZS* genes in several hosts (Suzuki et al., 2014). Thus, for example, overexpression of *RiRZS1* in tobacco hairy roots and various plant cell suspensions has allowed for raspberry ketone to be successfully produced with precursor feeding (5.5 mg/L) by bioconversion, using either 4-hydroxybenzalacetone or rhododenol as substrates (Hakkinen et al., 2015). Furthermore, previous work showed that engineered *E. coli* and *S. cerevisiae* successfully resulted in heterologous production of raspberry ketone; namely, 2.8 mg/L for *de novo* synthesis and 5–90 mg/L with precursor feeding (Lee et al., 2016; Wang et al., 2019). However, *de novo* synthesis of raspberry ketone without the need for precursor addition or culture medium has not yet been accomplished. In the present study, considerable levels, similar to the amounts found in ripe raspberries (i.e., 2.5–7.5 μg/g FW) of raspberry ketone, were found in the petals of *RpBAS*-*RiRZS1* overexpressing tobacco plants. However, trace amounts of raspberry ketone were detected in whole flowers but no raspberry ketone accumulation was observed in the leaves (Supplementary Table 2). This result suggests that the accumulation of raspberry ketone in different organs is cell-specific, whether the plant cells have developed the potential to produce and accumulate volatile compounds or not. For example, tobacco overexpressing the *limonene synthase* gene from *Perilla frutescens* produced a small amount (40 ng/g FW) of monocyclic monoterpene limonene, which has no hydroxy groups, while the expression of the same gene in *Eucalyptus camaldulensis* resulted in five-times more limonene (120 μg/g FW) than that accumulated in the wildtype (Ohara et al., 2003, Ohara et al., 2010).

The phenylpropanoid pathway provides the common precursor molecules for raspberry-ketone and anthocyanin synthesis (Fig. 1); CHS and RpBAS competitively use the same precursor, *p*-coumaroyl-CoA, in the phenylpropanoid pathway. In *RB*-OX plants, we observed that anthocyanin accumulation levels in the petal tissues of raspberry ketone-producing transgenic plants were 2- to 3-fold lower than those in the control and the transgenic plants with no raspberry-ketone production (Fig. 4A). This reduction in floral anthocyanin pigments suggests that the bottleneck of raspberry-ketone production in tobacco is the competition for available *p*-coumaroyl-CoA substrate between RpBAS and endogenous CHS for anthocyanin biosynthesis. Therefore, to enhance the production of raspberry ketone in the transgenic plants, it is necessary to increase the supply of substrates in the phenylpropanoid pathway. Previously, Xie et al. (2006) and Mitsunami et al. (2014) reported that the overexpression of *AtPAP1* in tobacco plants led to anthocyanin pigmentation through the transcriptional activation of structural genes in the anthocyanin biosynthetic pathway, including the one encoding PAL enzymes. For this reason, we cross-pollinated *RB*-OX plants with *PAP1*-OX plants, in which the phenylpropanoid pathway highly activates anthocyanin production in the whole plant. By genetic crossing, *AtPAP1* was introduced into the *RB*-OX lines and the co-expression of *RpBAS* and *RiRZS1* with *AtPAP1* in the transgenic lines resulted in increased accumulation of raspberry ketone and its glycosides up to 0.45 μg/g FW and 4.46 μg/g FW, respectively, in floral and vegetative tissues. Furthermore, this strategy revealed the accumulation of other phenylpropanoid derivatives, such as rhododenol and 4-hydroxybenzyl alcohol, which were produced in a transgenic line-specific manner in the *RB*-OX plants (#1) overexpressing only the *BAS* gene. Therefore, 4-hydroxybenzyl alcohol and rhododenol were formed from 4-hydroxybenzalacetone and raspberry ketone, respectively, through further metabolism by endogenous enzymes in tobacco plants (Fig. 1). For example, dihydroflavonol reductase (DFR), which belongs to a short chain dehydrogenase/reductase (SDR) superfamily, catalyzes the carbonyl reduction of dihydroflavonol into the corresponding alcohol (Lim et al., 2016). This enzyme or related unknown NADPH-dependent reductases may be involved in the formation of rhododenol from raspberry ketone in the *RB*-OX plants.

Our findings suggest that the transcriptional activation of biosynthetic genes by overexpression of *AtPAP1* via cross-pollination provided a better supply of pathway substrates to the phenylpropanoid pathway, especially in leaves, thereby increasing the biosynthesis of raspberry ketone and its related compounds. The biosynthesis of C_6_–C_1_ aromatic volatiles, including benzyl alcohol, from cinnamic acid has been reported in petunia (*Petunia hybrida*) and black cottonwood (*Populus trichocarpa*), and the peroxisomal β-oxidative pathway contributes to the shortening of the propyl side chain by two carbons in phenylpropanoids (C_6_–C_3_) (Lackus et al., 2021; Widhalm and Dudareva, 2015). Based on this information, it is possible that the β-oxidative pathway potentially mediates the C_2_ shortening of the propyl side chain of cinnamic acid to yield benzyl alcohol over several enzymatic steps in the *RB*-OX and the *RB*-OX × *PAP1*-OX plants. As raspberry ketone and benzyl alcohol share a precursor in the general phenylpropanoid pathway, it is conceivable that the metabolic flux leading to the formation of benzyl alcohol is affected by the overexpression of *RpBAS* and *RiRZS1* with *AtPAP1*, resulting in a change in benzyl alcohol contents in the transgenic lines.

Similarly, in the *PAP1*-OX plants, to increase the supply of pathway substrate *p*-coumaroyl-CoA to the phenylpropanoid pathway, we performed a metabolic switching by suppressing *CHS* in *RB*-OX plants. This was achieved via cross-pollination with *CHSir*-OX plants, which normally produce white flowers owing to the low levels of anthocyanin production in the transgenic plants. Consistently, *RB*-OX plants with an increased availability of the substrate for RiRZS1 by the suppression of *CHS* showed enhanced production of raspberry-ketone derivatives in the flower tissues (Fig. 7). This result further supports the idea that an increased supply of endogenous substrates into the phenylpropanoid pathway is crucial for the efficient production of raspberry ketone in plants. It is assumed that the cross-pollinated plants of RBP and RBC produce higher amounts of raspberry ketone than RBP plants or RBC plants; nevertheless, further research is required to confirm the levels of productivity.

In vegetative tissues of various plant species, volatile compounds are in some cases accumulated as glycosides (Song et al., 2018). Most raspberry-ketone compounds produced in transgenic plants are conjugated with a glycoside moiety, thus forming raspberry-ketone glycosides. Although we did not examine the localization of raspberry-ketone glycosides, it might be accumulated in the vacuoles of plant cells, similar to other classes of secondary metabolites (Takanashi et al., 2014; Can'ani et al., 2017). In the present study, raspberry-ketone aglycones were occasionally detected in trace amounts (i.e., 0.4–0.8 μg/g FW). In general, the glycosylated forms were observed in the *RB*-OX plants that were cross-pollinated with *PAP1*-OX plants and *CHSir*-OX plants (i.e., 2.5–5.6 μg/g FW). The finding suggests that raspberry ketone produced in transgenic plants are rapidly converted to the corresponding glycosides by the action of endogenous enzymes, such as UDP-glycosyltransferases. In fact, emission of free raspberry-ketone aglycones to head-space was not detected by head-space GC–MS analysis, whereas volatile terpenoids including linalool and caryophyllene were detected (Supplementary Fig. 6). These results may suggest that tobacco flowers do not have emission systems for raspberry ketones via simple diffusion or transporters and lipid transfer proteins.

In conclusion, the co-expression of *RpBAS* and *RiRZS1* with *AtPAP1* overexpression and *CHS* suppression are capable of enhancing the metabolic flux of intermediate metabolites, which are the potential substrates for raspberry ketone biosynthesis. Ultimately, such a strategy, in combination with the glycosylating capacity of plant cells, offer a means to enhance the accumulation of raspberry-ketone derivatives in transgenic tobacco plants, which would be available as sustainable bioresources for production of plant specialized metabolites for application in the cosmetic and food industries.

## Author contribution

Takao Koeduka: Conceptualization, Investigation, Validation, Supervision, Writing-Original Draft, Writing-review & editing, Funding acquisition. Sachiho Takarada: Investigation, Data curation. Koya Fujii: Investigation, Data curation. Akifumi Sugiyama: Writing-review & editing. Kazufumi Yazaki: Writing-review & editing. Masahiro Nishihara: Resources, Writing-review & editing. Kenji Matsui: Writing-review & editing.

## Funding

This work was partially supported by a Grant-in-Aid for Scientific Research from the 10.13039/501100001691Japan Society for the Promotion of Science (JSPS; no. 20K05840 to T.K.) and by a research grant from 10.13039/501100009405RISH Mission Research, Kyoto University.

## Declaration of competing interest

The authors declare that they have no known competing financial interests or personal relationships that could have appeared to influence the work reported in this paper.
