# Supplementary material for: Production of raspberry ketone by redirecting the metabolic flux to the phenylpropanoid pathway in tobacco plants
Source: Metab Eng Commun. 2021 Jul 27;13:e00180. doi: 10.1016/j.mec.2021.e00180 (PMC8346687; doi:10.1016/j.mec.2021.e00180)
Supplement: Multimedia component 1 [file mmc1.pdf]

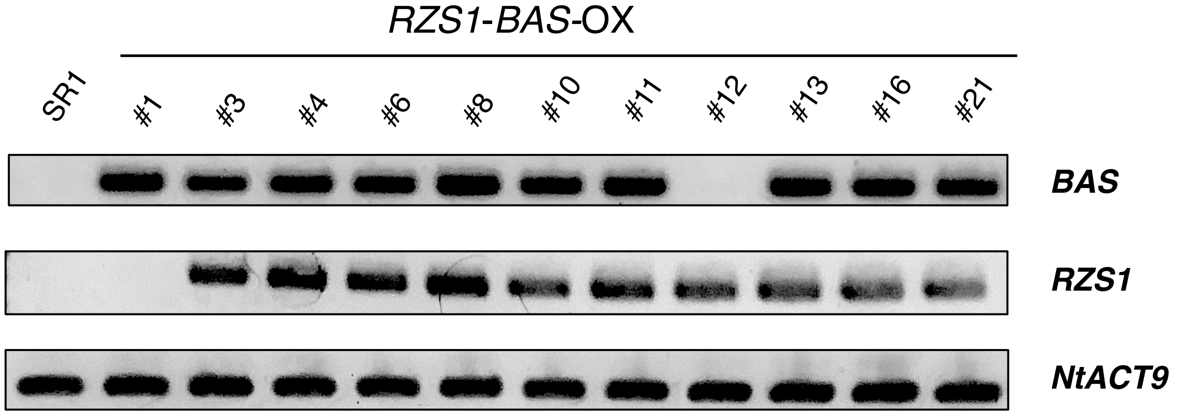

Figure S1 Genomic-DNA PCR analysis of *RZS1* and *BAS* genes in transgenic tobacco lines. The amplification of *BAS*, *RZS1*, and *NtACT9* were analyzed using genomic-DNA PCR. *RZS1*, *raspberry ketone/zingerone synthase 1*; *BAS*, *benzalacetone synthase*; *NtACT9*, *N. tabacum Actin 9*. *NtACT9* was used as an internal control.

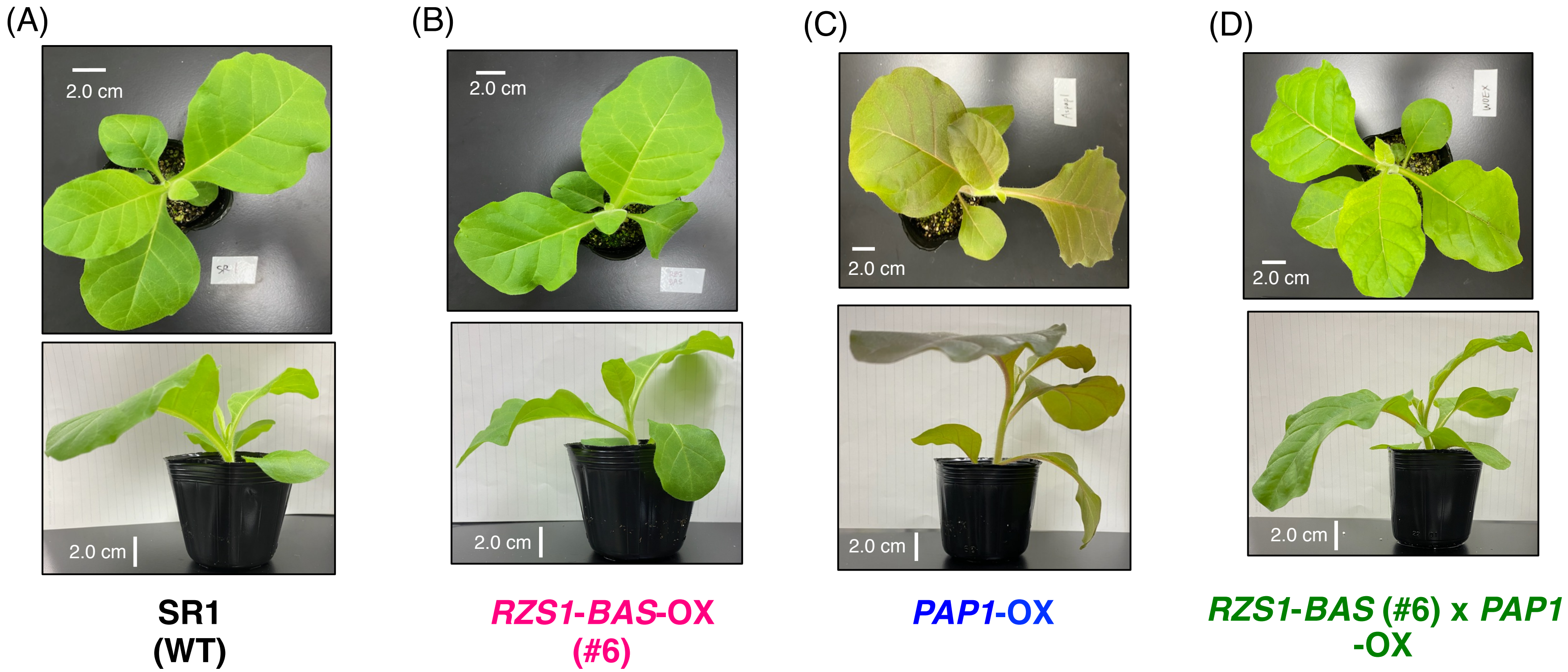

Figure S2 Phenotype of engineered transgenic tobacco plants (T_2 generation). (A) Wild-type tobacco cv. SR1. (B) *RZS1-BAS* overexpressing transgenic tobacco. (C) *PAP1* overexpressing transgenic tobacco. (D) *RZS1-BAS* and *PAP1* overexpressing transgenic tobacco. All plants were grown in culture room for 42 days. Scale bar indicates 2.0 cm. *RZS1*, *raspberry ketone/zingerone synthase 1*; *BAS*, *benzalacetone synthase*; *PAP1*, *production of anthocyanin pigment 1*.

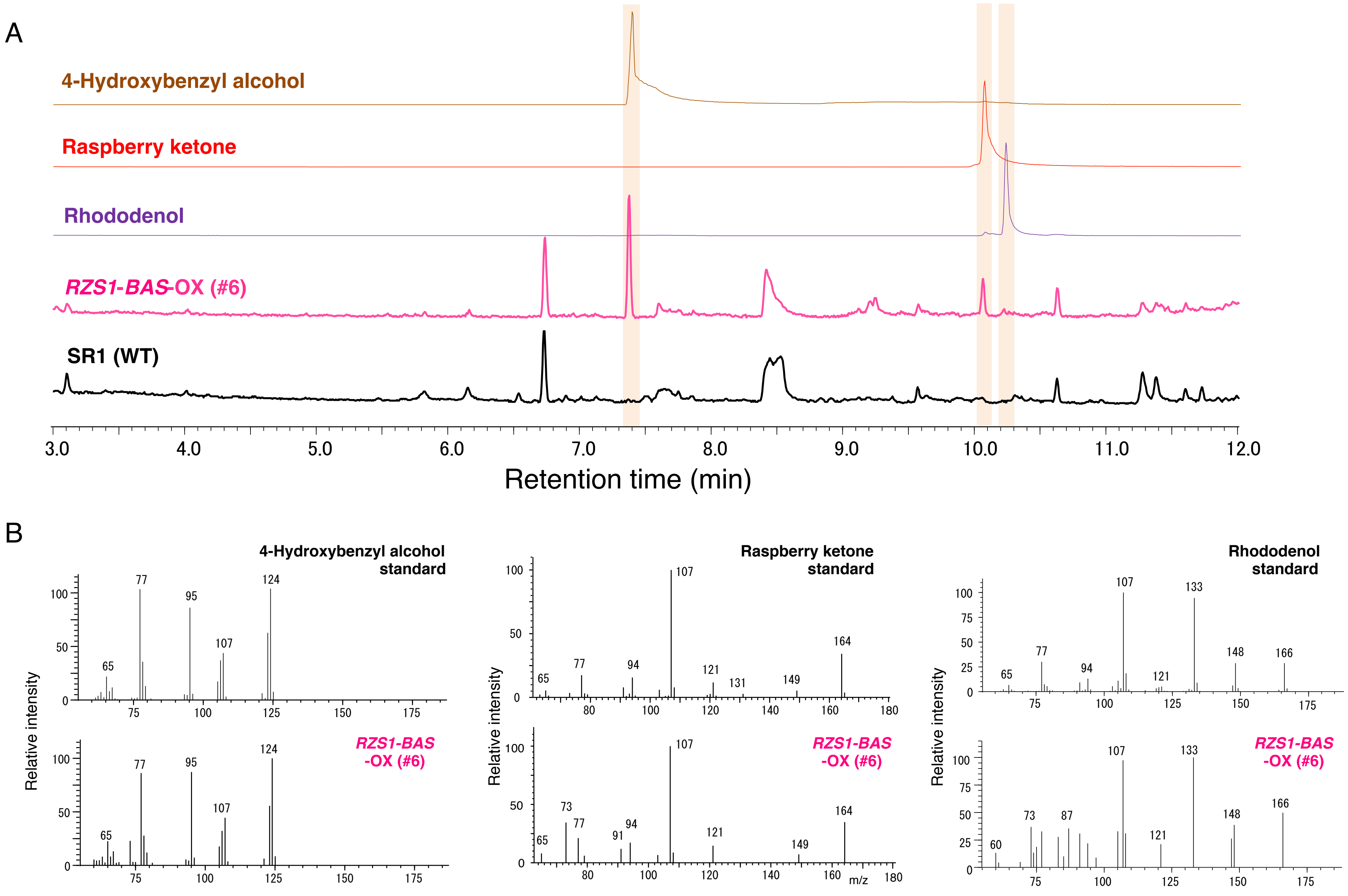

Figure S3 GC–MS analysis of volatile compounds extracted from *RZS1-BAS* overexpressing transgenic tobacco petals. (A) GC–MS chromatograms of petal extracts from *RZS1-BAS*-OX tobacco (line #6). (B) Mass spectrum of raspberry ketone and its derivatives. The peaks of each volatile compound were identified by comparing with the retention time and mass spectrum of authentic standards. Mass spectra of authentic standard (upper panels) and peaks detected from petal extracts (lower panels) are shown.

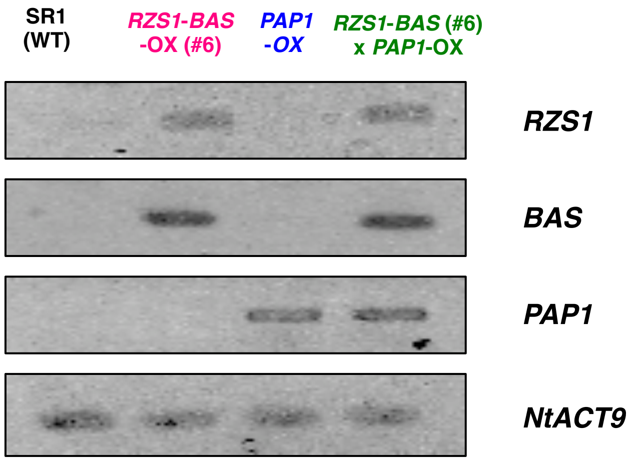

Figure S4 Expression of genes for raspberry-ketone biosynthesis and transcription factors PAP1. The transcript levels of *RiRZS1*, *RpBAS*, and *AtPAP1* in three different transgenic tobacco lines were analyzed using semi-quantitative RT-PCR. *RZS1*, *raspberry ketone/zingerone synthase 1*; *BAS*, *benzalacetone synthase*; *PAP1*, *production of anthocyanin pigment 1*; *NtACT9*, *N. tabacum Actin 9*. *NtACT9* was used as an internal control.

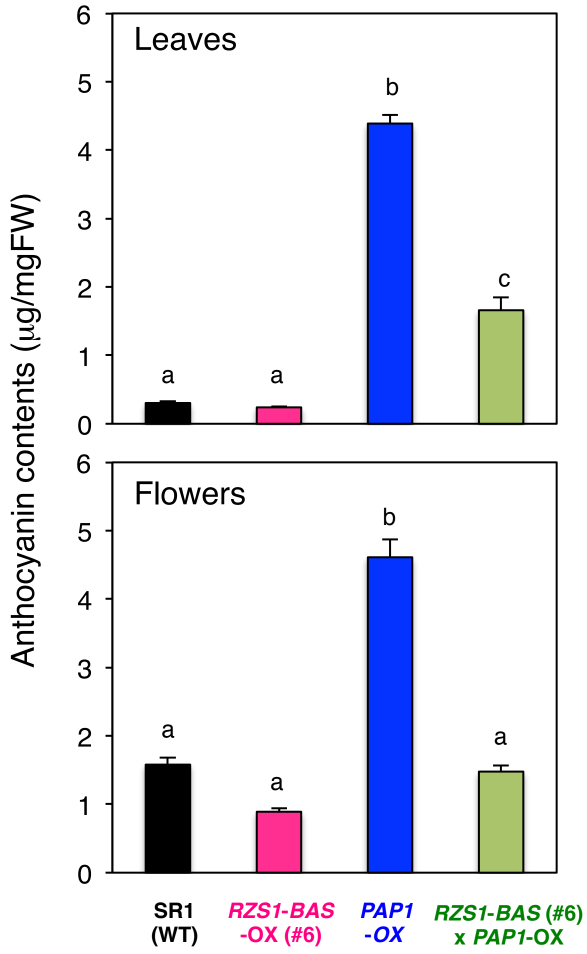

Figure S5 Accumulation of anthocyanin pigments in three different transgenic tobacco lines. Anthocyanin contents were determined by measuring absorbance spectrophotometrically at 530 nm, as described in "Materials and Methods". Three flowers were collected from independent transgenic tobacco plants and the values indicate the averages ± standard deviations for three biological replicates. Significant differences indicated by different lowercase letters were identified using Tukey's HSD tests after one-way ANOVA (P <0.05).

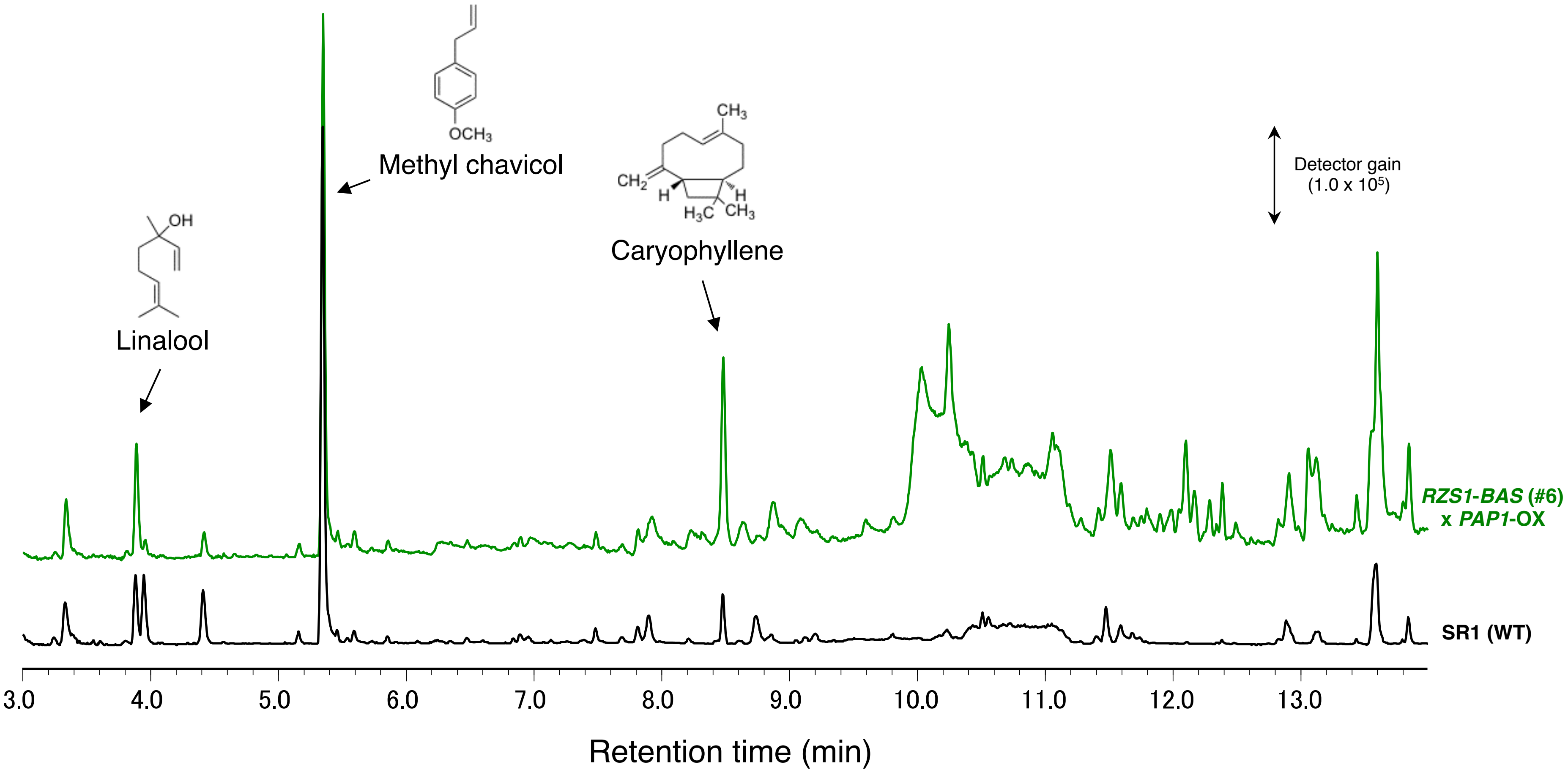

Figure S6 Chromatogram obtained with headspace SPME–GC–MS analysis of volatile compounds emitted from *RZS1-BAS* and *PAP1* overexpressing transgenic tobacco flowers.
