# Supplementary material for: Production of raspberry ketone by redirecting the metabolic flux to the phenylpropanoid pathway in tobacco plants
Source: Metab Eng Commun. 2021 Jul 27;13:e00180. doi: 10.1016/j.mec.2021.e00180 (PMC8346687; doi:10.1016/j.mec.2021.e00180)
Supplement: Multimedia component 2 [file mmc2.pdf]

Table S1

Oligonucleotide sequences used for the semi-quantitative RT-PCR in this study

Direction	Primer sequence (5′→3′)
**Primers used for semi-quantitative RT-PCR**	
**For *NtACT9***	
Forward	CTATTCTCCGCTTTGGACTTGGCA
Reverse	AGGACCTCAGGACAACGGAAACG
**For *RpBAS***	
Forward	CATATGGCTACTGAAGAGATGAAG
Reverse	TGAGTCTCAGATGGTCCCCTGAA
**For *RiRZS1***	
Forward	AATAATCGGTTATGGAGTGGCTAGA
Reverse	TCCTTAGAGCAGCATCCAGGTCA
**For *AtPAP1***	
Forward	AACTCTAGAATGGAGGGTTCGTCCAAAGG
Reverse	TTCCGAGCTCCTAATCAAATTTCACAGTCTCT
**Primers used for quantitative RT-PCR**	
**For *NtEF1α***	
Forward	CCACACCTCCCACATTGCTGTCA
Reverse	CGCATGTCCCTCACAGCAAAAC
**For *RpBAS***	
Forward	GGGTGCTAGGGTGTTGATT
Reverse	CAGCACCATCTCCAAGGATAG
**For *RiRZS1***	
Forward	CACTGATGTGCCTCTCTCTTAC
Reverse	GCCAACAAGCTGACCTACT
**Primers used for full-length *RiRZS1* and *RpBAS* cloning**	
**For *RpBAS***	
Forward	CATATGGCTACTGAAGAGATGAAG
Reverse	TCAAGAGATCACTGGCACAG
**For *RiRZS1***	
Forward	GCGCGGCAGCCATATGGCGAGTGGTGGAGAAATG
Reverse	ACGGAGCTCGAATTCCTCGAGTCACTCTCTGGAAACAACCAC
**Primers used for InFusion cloning**	
**For binary vector constructs**	
Forward	TGCGGCCGCTGGATCGTCCCCAGATTAGCCTTTTC
Reverse	CCATGATTACGAATTAATTCCTTATCTTTAATCATATTC

Table S2. Volatile benzenoids and those glycosides in leaves of transgenic tobacco.

Compound	**SR1 (WT)**		Transgenic lines: ***RZS1-BAS* -OX (#6)**		***PAP1* -OX**		***RZS1-BAS* (#6) x *PAP1* -OX**	
	aglycone	glycosides	aglycone	glycosides	aglycone	glycosides	aglycone	glycosides
Benzyl alcohol	n.d.	1.42 ± 0.15	n.d.	1.62 ± 0.20	0.65 ± 0.04	1.55 ± 0.54	1.02 ± 0.05	2.94 ± 0.24
2-Phenylethanol	n.d.	0.32 ± 0.07	n.d.	0.46 ± 0.07	n.d.	0.11 ± 0.03	0.45 ± 0.03	0.41 ± 0.05
Raspberry ketone	n.d.	n.d.	n.d.	n.d.	n.d.	n.d.	n.d.	2.24 ± 0.18
Rhododenol	n.d.	n.d.	n.d.	n.d.	n.d.	n.d.	n.d.	2.29 ± 0.18

(μg/gFW)

Table S3. Volatile benzenoids and those glycosides in flowers of transgenic tobacco.

Compound	**SR1 (WT)**		Transgenic lines: ***RZS1-BAS* -OX (#6)**		***PAP1* -OX**		***RZS1-BAS* (#6) x *PAP1* -OX**	
	aglycone	glycosides	aglycone	glycosides	aglycone	glycosides	aglycone	glycosides
Benzyl alcohol	1.15 ± 0.07	4.56 ± 2.95	0.98 ± 0.04	0.50 ± 0.03	0.38 ± 0.01	0.15 ± 0.02	0.23 ± 0.02	0.23 ± 0.09
4-Hydroxybenzyl alcohol	n.d.	n.d.	n.d.	n.d.	n.d.	n.d.	0.12 ± 0.03	0.76 ± 0.17
Raspberry ketone	n.d.	n.d.	n.d.	2.38 ± 0.35	n.d.	n.d.	0.45 ± 0.04	4.46 ± 0.21
Rhododenol	n.d.	n.d.	n.d.	0.60 ± 0.09	n.d.	n.d.	n.d.	1.78 ± 0.04

(μg/gFW)
